# Effects of Nitro-Oxidative Stress on Biomolecules: Part 2—Reactive Molecular Dynamics Simulations

**DOI:** 10.3390/biom15070952

**Published:** 2025-06-30

**Authors:** Zhaonan Chai, Yawei Feng, Tong Zhao, Xiaolong Wang, Maksudbek Yusupov, Maryam Ghasemitarei, Tayebeh Ghorbi, Annemie Bogaerts, Yuantao Zhang

**Affiliations:** 1 School of Electrical Engineering, Shandong University, Jinan 250061, China; 2 Institute of Fundamental and Applied Research, National Research University TIIAME, Tashkent 100000, Uzbekistan; 3 School of Engineering, New Uzbekistan University, Tashkent 100000, Uzbekistan; 4 Department of Applied Physics, Aalto University, FI-00076 Espoo, Finland; 5 Laboratory of Experimental Biophysics, Centre for Advanced Technologies, Tashkent 100174, Uzbekistan; 6 Research Group PLASMANT, Department of Chemistry, University of Antwerp, 2610 Antwerp, Belgium

**Keywords:** cold atmospheric plasma, biological tissues, environmental toxins, reactive MD simulation, reactive oxygen species, 52.80Pi, 52.65.Kj, 52.77.Fv

## Abstract

In this review article, statistical mechanisms of oxidative modification reactions in various organic compounds under the influence of reactive oxygen species (ROS) generated by cold atmospheric plasma (CAP) are investigated and analyzed based on reactive molecular dynamics (MD) simulations. As an efficient and hygienic advanced oxidation technology, CAP demonstrates tremendous potential in fields such as biomedicine and environmental protection. Through simulations, this paper provides a detailed analysis of the interaction mechanisms between ROS and components of biological tissues and environmental toxins. In this paper, we review the reactions involving four major ROS (OH radicals, O atoms, $O_{3}$ molecules, and $H_{2}O_{2}$ molecules) and organic compounds, including proteins, DNA, polysaccharides, fatty acids, antibiotics, and mycotoxins. Atomic-level analysis reveals various oxidative modification reactions induced by ROS and their resulting products, including dehydrogenation reactions, bond-formation reactions, oxygen-addition reactions, and bond-cleavage reactions. Additionally, the study elucidates the role of active functional groups in various organic compounds, the presence of special elements, and the specific reactive nature of $H_{2}O_{2}$. Furthermore, the influence of different ROS species and concentrations on reaction types is explored, aiming to provide a solid theoretical foundation for the application of CAP technology in biomedicine and environmental remediation.

## 1. Introduction

For an extended period, cold atmospheric plasma (CAP) has demonstrated tremendous potential across diverse application domains, including biomedicine and environmental protection [1,2,3,4]. It has been widely utilized in medical device sterilization [5,6], wound healing [7], blood coagulation and hemostasis [8,9], food industry packaging [10], and environmental toxin degradation [11], among other scenarios. As a highly efficient and hygienic advanced oxidation technology, CAP has the capability to generate high-density plasma (ranging from 10^11^ to 10^16^ cm^−3^) without requiring complex vacuum equipment while maintaining a low-temperature environment [12]. These attributes contribute to a reduction in equipment costs for CAP generation and facilitate large-scale, continuous processing.

Notably, CAP not only exhibits remarkable bactericidal efficacy but also has the unique advantage of easily penetrating narrow and confined spaces, enabling rapid, contactless disinfection, even within minute gaps or minimally invasive small openings [13,14]. Moreover, its near-room-temperature operating conditions significantly expand its applicability to temperature-sensitive materials, further driving its development in biomedicine, environmental governance, and food and agriculture. The versatility and efficacy of CAP in these fields underscore its potential as a pioneering technology with wide-ranging implications for future research and practical applications.

In the realm of medicine, plasma technology has demonstrated promising outcomes in tissue disinfection, wound modulation and treatment, and respiratory inflammation management and has even opened new avenues of hope for cancer therapy [15,16,17,18,19,20]. Numerous studies have substantiated the efficacy of plasma in various applications, including bactericidal activity, biofilm eradication, surface disinfection, decontamination processes, protein removal, and blood coagulation [21,22,23,24,25,26]. Moreover, plasma holds immense potential for further advancements in healthcare, including antifungal therapies, dental care, skin disease rehabilitation, chronic wound management, and medical aesthetics [27,28,29,30,31,32]. Particularly in the context of cancer treatment, CAP has exhibited properties that induce apoptosis in cancer cells and inhibit tumor cell growth, thereby shedding new light on the ongoing battle against cancer [33].

On the other hand, the overuse of medical treatments has contributed to the infiltration of organic pollutants, such as various drugs, antibiotics, and mycotoxins produced by fungal growth, into soil, water sources, air, and even the food chain [34,35,36,37,38,39]. These pollutants, due to their covert and persistent nature, form a complex and widespread pollution network [40]. The degradation of drug contaminants in the natural environment is particularly challenging, and residual pharmaceutical compounds pose direct threats to wildlife survival and reproduction, and they disrupt ecological balance, enhance bacterial resistance, and seriously endanger food safety [41]. Drug pollutants can have profound impacts on human health either through the ingestion of contaminated food or via cumulative effects along the food chain, leading to issues such as endocrine disruption, impaired immune function, neurotoxicity, and an increased risk of cancer [42]. Collectively, these pollutants present a severe challenge to both ecosystems and human health. Notably, the generation of a substantial number of reactive oxygen and nitrogen species (RONS) and high-energy electrons by plasma enables chemical reactions with pollutant molecules in the environment. This process facilitates effective degradation and harmless transformation of pollutants, highlighting the potential of plasma technology in addressing environmental contamination [43,44,45,46].

Despite significant progress and optimization in research on plasma degradation of various environmental pollutants compared to traditional treatment technologies, studies in this field remain primarily focused on assessing degradation efficiency and exploring the influence of plasma discharge parameters (such as discharge duration, gas composition, and discharge energy) on this efficiency [47]. These areas of focus are largely constrained by current limitations in experimental diagnostic techniques. Consequently, there is a relative lack of exploration into the underlying microscopic mechanisms governing these processes.

The identification of degradation products and pathways predominantly relies on mass spectrometry and chromatography analysis, often supplemented by hypothetical inferences [48]. Similarly, in the biomedical field, although plasma applications have demonstrated promising efficacy in both experimental and clinical settings, the mechanisms underlying CAP interactions with various tissues remain poorly understood [49]. To address these knowledge gaps, employing appropriate molecular simulation techniques can provide valuable insights. These techniques can visually demonstrate the formation and cleavage of chemical bonds during the interaction between plasma-generated RONS and biological tissues or environmental pollutants. By tracing the entire reaction process from initial reactants to final products, simulations can elucidate the microscopic mechanisms by which plasma exerts its effects, including the pathogen eradication and environmental pollutant degradation.

Molecular simulation, a powerful tool for investigating the structural and functional properties of molecular systems, is widely applied in chemistry, biology, and materials science [50,51,52], functioning as a “computational experiment”. Within this domain, molecular dynamics (MD) simulation emerges as a technique that determines the positional coordinates of molecules by solving the equations of motion derived from classical mechanics [53,54,55]. It further calculates the intermolecular forces to determine the dynamic evolution trajectory of the system under study. By leveraging precise force field parameters, this methodology can provide an accurate depiction of the intricacies of intermolecular interactions, evaluate the cleavage and formation of chemical bonds, and offer microscopic-level insights into reaction mechanisms within chemical systems.

The purpose of this paper is to provide a specialized mechanistic/theoretical review on reactive MD simulations to explore oxidative modification reactions of diverse organic compounds affected by reactive oxygen species (ROS) from CAP. It delves into atomic-level reaction mechanisms, the role of functional groups, and ROS impacts, offering unique insights for CAP applications.This article systematically elaborates on the principles and characteristics of the reactive MD simulation, with a particular focus on the Reax force field (ReaxFF) employed in these simulations (detailed in Section 2). Building upon this foundation, the study explores the intricate interaction mechanisms between plasma-generated ROS and various components of biological tissues and environmental toxins (comprehensive analysis in Section 3). Through rigorous atomic-level analysis, this research unveils the diverse types of oxidative modification reactions and the corresponding products induced by ROS in various organic compounds. Furthermore, the study conducts a comparative analysis of the impacts of different reactive species and examines how ROS concentration influences reaction types. The objective is to compile and summarize oxidation reactions comprehensively, ultimately establishing a universal set of reaction laws. In Section 4, the article presents a comprehensive synthesis of oxidative modifications induced by plasma-generated ROS and highlights the promising applications of CAP technology in biomedical and environmental remediation. These findings not only establish a solid theoretical foundation for the industrial application of CAP technology but also provide scientific and theoretical guidance for understanding the mechanisms of ROS processing generated by plasma.

## 2. Description of Numerical Methods

### 2.1. Reactive Molecular Dynamics Simulation

Advances in computer technology have led to the emergence of molecular simulation techniques. As a “computational experimental” approach that relies on programmatic algorithms, molecular simulation serves as a complementary method to theoretical analysis and experimental observations, providing a third pathway for exploring the structural features and behavioral patterns of molecular systems [56]. This field encompasses several branches, including Monte Carlo simulation, molecular mechanics simulation, molecular dynamics simulation, and quantum mechanics simulation.

Monte Carlo methods involve random sampling within the space defined by particle position coordinates, without considering particle momenta. As a time-independent method, its limitation lies in its inability to capture dynamic evolution within the system. Molecular mechanics simulation is primarily used to determine the optimal geometric configuration of molecules and often precedes molecular dynamics simulation, ensuring the rationality of the initial structure and facilitating the smooth progression of the simulation [57,58]. MD simulation, on the other hand, incorporates intermolecular interaction forces and iteratively updates molecular coordinates and momenta based on Newton’s equations of motion. This approach enables the acquisition of dynamic characteristics that evolve over time. Quantum mechanics simulation offers high accuracy and closely aligns with experimental results. However, it demands immense computational resources, requires high-specification equipment, and is constrained by spatial (limited to a few hundred atoms) and temporal (on the order of picoseconds) scales of the simulated molecules. Consequently, it is seldom applied to macromolecular systems [59].

MD simulation offers a unique avenue for unveiling atomic-scale information that is often inaccessible through experiments. This granular atomic-scale data provides profound insights into the macroscopic properties observed in experimental settings. Within the theoretical framework of statistical mechanics, the interpretation of these macroscopic system properties necessitates a comprehensive consideration of the underlying microscopic states, thereby establishing a theoretical foundation for the application of MD simulation [60]. Since statistical mechanics serves as the fundamental theory behind MD simulations, and these simulations must be conducted under specific conditions, selecting an appropriate statistical mechanics ensemble is paramount to the success of the simulation. From a statistical mechanics standpoint, macroscopic properties, such as pressure and density, are statistical averages of the system’s quantum states. Ensembles are employed to evaluate the cumulative contributions of all quantum states across a large number of studied systems to derive specific macroscopic properties. In practical terms, commonly used simulation ensembles include the microcanonical ensemble (NVE), canonical ensemble (NVT), isothermal–isobaric ensemble (NPT), and grand canonical ensemble (µVT). Each of these ensembles adheres to distinct constraints, involving parameters such as particle number (N), volume (V), energy (E), temperature (T), pressure (P), and chemical potential (µ). Specifically, the microcanonical ensemble (NVE) simulates an isolated system that does not exchange energy with the external environment. The canonical ensemble (NVT) models a system embedded within a larger system maintained at a constant temperature. The isothermal–isobaric ensemble (NPT) extends this by incorporating constant pressure conditions. Lastly, the grand canonical ensemble (µVT) is designed for open systems where the number of particles varies. The NVT is widely applied in the investigation of interaction between CAP and biomolecules, which can clearly unveil the reaction pathways in plasma medicine [19,61,62,63].

### 2.2. Reax Force Field

Building on the theoretical foundation of molecular dynamics, reactive MD simulation has been developed to provide deep insights into the processes of chemical bond breaking and formation, as well as to simulate chemical reactions within molecular systems. The cornerstone of this technology lies in the judicious selection of the potential function, commonly referred to as the molecular force field, which consists of both the functional form and the associated force field parameters. These parameters must be carefully chosen based on the distinct characteristics of the specific molecules and atoms under investigation, while the functional form of the potential function exhibits a certain degree of universality.

In the context of chemical reaction studies, reactive molecular force fields play a crucial role. Among these, bond-order-dependent potential functions, such as Brenner, REBO, and ReaxFF, are particularly significant. Notably, ReaxFF stands out as the most widely utilized force field in this domain [64]. Developed by the Van Duin team, ReaxFF was originally designed to elucidate complex chemical reactions in hydrocarbons. It demonstrates remarkable transferability across the periodic table, making it highly versatile for studying molecular systems containing various compounds [65]. Through rigorous comparisons with quantum simulations and experimental data, this force field has been meticulously parameterized to capture atomic properties, bonding states, angles, torsional characteristics, and interparticle interactions within a system. As a result, it achieves simulation results that closely approximate the accuracy of quantum mechanics calculations while maintaining relatively low computational costs. This advancement has significantly expanded the scale of simultble systems, particularly in terms of the number of particles. Consequently, reactive MD simulation, particularly using ReaxFF, has found widespread application across various chemical reaction fields, including combustion, pyrolysis, catalysis, supercritical states, battery technology, electrical engineering, building materials, tribology, biopharmaceuticals, perovskite materials, semiconductors, and energy materials [66,67,68].

In the context of bond-order-dependent force fields, ReaxFF effectively accounts for the interaction between bond distance and bond order, as well as the correlation between bond order and bond energy. The bond order is dynamically determined based on the instantaneous distance between atoms, with updates occurring at each simulation step. This approach enables the accurate representation of chemical bond formation and cleavage processes. Moreover, bond order plays a crucial role in determining the system’s energy, which serves as a basis for analyzing intermolecular interaction forces through energy computations. The energy expression for the system can be concisely outlined as follows:

(1) $$E_{system}=E_{bond}+E_{over}+E_{angle}+E_{tors}+E_{vdWaals}+E_{coulomb}+E_{specific}$$

In Equation (1), $E_{bond}$ is expressed as a continuous function of interatomic distances, providing a quantitative measure of the energy associated with bonding interactions between atoms. The terms $E_{angle}$ and $E_{tors}$ correspond to the energy contributions from three-body valence angle strain and four-body torsional angle strain, respectively. $E_{over}$ functions as an energy penalty term, preventing atoms from exceeding valence bond rules through over-coordination (e.g., a string energy penalty is applied when a carbon atom forms more than four bonds). The contributions of electrostatic interactions and dispersion energy among all atoms are represented by $E_{vdWaals}$ and $E_{coulomb}$, respectively. To account for non-bonded interactions within dynamically connected systems, van der Waals and Coulomb forces are incorporated. These forces are independently computed for any pair of atoms, remaining unaffected by connectivity and bond order. Furthermore, they are subject to shielding effects at short distances and asymptotically converge to a constant as the distance approaches zero. Lastly, $E_{specific}$ represents specific energy terms that may be required for particular research systems. These terms are typically excluded from the standard energy expression, unless they are essential for investigating specific properties, such as lone-pair electron effects, conjugation effects, hydrogen bonding, and C2 corrections [65]. In this review, we focus on the interactions of CAP and biomolecules with only several types of ROS considered, and all simulations were performed using the ReaxFF force field, which is particularly suited for modeling the formation and breaking of chemical bonds.

### 2.3. Construction of Reactive MD Simulations

Here we explain the basics of reactive MD simulations, utilizing ReaxFF as the reactive force field. It conducts a statistical analysis and explores the interaction mechanisms between the principal ROS generated by plasma and the constituents of biological tissues, as well as toxins and drug residues found in the environment. The objective is to provide an intuitive understanding of oxidative modification reaction types and the resultant reaction products of organic substances induced by plasma-generated ROS. The general procedure for model construction and the corresponding parameter settings are outlined below.

The initial phase of the simulation involves the construction of models for the objects of study. We focus here on developing simulation models for organic substances and four distinct types of ROS. These models can either be sourced from existing databases or constructed manually. Given the relatively low molecular weight of several organic substances and the straightforward structure of the reactive species, manual construction of model systems was chosen here. Specifically, atoms and ring structures were meticulously added in accordance with the actual structural configurations of each organic substance and reactive species, and appropriate chemical bonds were selected to link them together. However, manually constructed molecular models may exhibit deviations in structural parameters, such as bond lengths, bond angles, and dihedral angles, from their real values. Therefore, further structural adjustments are necessary to correct any inaccuracies introduced during the manual construction process.

Following the initial construction and preliminary adjustment of the molecular structures, they must undergo two crucial optimization stages to meet the prerequisites for reactive MD simulations. The first stage involves geometry optimization, which is essentially an energy minimization process. Through the iterative application of energy minimization algorithms, molecular structures are meticulously refined to eliminate high-energy conformations arising from unreasonable structural configurations, thereby ensuring the attainment of geometrically favorable conformations and establishing a robust foundation for the seamless progression of subsequent simulations. For the diverse molecular structures examined here, a cascade strategy incorporating three classic algorithms—namely, the steepest descent method, conjugate gradient method, and Quasi-Newton methods (e.g., BFGS) was employed to efficiently achieve the energy minimization objective. This process utilizes the COMPASS force field and imposes stringent convergence criteria, requiring the energy to be below 0.001 kcal/mol and the force below 0.5 kcal/mol/Å [69,70].

However, the molecular structures obtained after geometry optimization do not fully meet the practical requirements of reactive MD simulations, primarily because geometry optimization focuses solely on mathematical processing. To ensure that the simulation system better reflects real conditions, it is imperative to incorporate thermodynamic factors, such as temperature and pressure. As a result, kinetic optimization is introduced as a complement to geometric optimization. In this paper, the NVT ensemble was chosen as the thermodynamic ensemble for each molecular structure, ensuring that the number of particles (N), volume (V), and temperature (T) within the system remain constant throughout the simulation. Specifically, the temperature was set to 300 K, and the Berendsen method, also known as the thermal bath coupling technique, was employed for temperature control. This method maintains the specified temperature conditions by facilitating coupling between the system and the external environment.

Following the optimization of the organic substance molecular models, they were encapsulated alongside the ROS models relevant to the reaction, including OH radicals, O atoms, $O_{3}$ molecules, and $H_{2}O_{2}$ molecules, into a reaction unit cell. This unit cell was constructed as a three-dimensional periodic structure comprising multiple components. The COMPASS general force field was utilized for this encapsulation step. The reaction unit cell adopts a cubic structure, with a vector length (equivalent to the side length of the cubic unit cell) typically set to tens of angstroms. This length is constrained by factors such as the system volume, density, and computational load. Given the three-dimensional periodic nature of the reaction unit cell, periodic boundary conditions (PBCs) are typically imposed to constrain the system. Under these conditions, when a particle moves out of the unit cell, it is replaced by another particle entering from the opposite direction, ensuring that the total number of particles within the unit cell remains constant. This approach effectively eliminates boundary effects, making the simulation more representative of real-world conditions.

### 2.4. Parameter Setting

After the construction of the reaction unit cell, it is necessary to select key parameters for reactive MD simulations, including the statistical mechanical ensemble, reaction time, time step, and system temperature. Of particular importance is the selection of the force field, which is a critical step in the process. ReaxFF is well suited for reactive MD simulations. This force field is distinguished by its versatility, with its parameter set comprehensively covering both organic and inorganic systems. Specifically, it encompasses the chemical elements present in various biological tissues, as well as environmental toxins and plasma-generated ROS, making it highly suitable for simulating their interactions.

**Figure 1:**
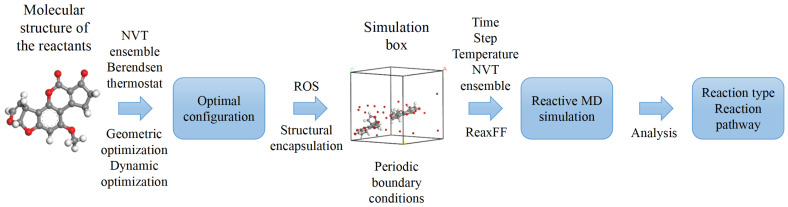
Flowchart of a complete reactive molecular dynamics (MD) simulation.

Once the force field has been determined, it is necessary to set detailed parameters to regulate the computational process of the simulation system. The NVT (canonical) ensemble is typically selected to ensure that the number of particles, system volume, and temperature remain constant throughout the simulation. To approximate real-world conditions, the simulation temperature is set to room temperature, specifically 300 K. The time step is a crucial parameter in the integration algorithm, as it directly influences computational efficiency and accuracy. While a larger time step can expedite calculations, it may also result in increased errors or even computational instability due to excessive energy deviations. Based on the total reaction duration, a time step of 0.1 fs is considered a reasonable value. The total simulation duration is determined through multiple simulations of different reaction systems, with a few hundred picoseconds generally deemed sufficient for the reactive MD simulation tasks presented in this paper. Additionally, an equilibration calculation of 10-50 ps is conducted prior to the formal simulation to ensure that the system reaches a stable state. The complete flowchart for a reactive MD simulation is illustrated in Figure 1.

The model construction for the various biological tissues and environmental toxins discussed in this review follows consistent simulation conditions. The constructed reaction unit contains one or two biomolecules to interact with ROS, with doses ranging from 5 to 50, given in Figure 1. The unit cell adopts a cubic structure with dimensions of 20 Å × 20 Å × 20 Å. By adjusting the number of ROS within the reaction unit cell, we investigate the impact of dose effects on molecular degradation. All simulation experiments are performed under the NVT ensemble, with a 50 ps relaxation period to achieve equilibrium. Both the relaxation and reaction phases are maintained at a constant temperature of 300 K. To ensure simulation accuracy, each simulation is set for a reaction time of 300 ps, utilizing a fine time step of 0.1 fs to comprehensively capture the details of oxidative modification reactions. To mitigate the influence of random errors and obtain a more comprehensive understanding of the reaction mechanisms, each set of simulations is repeated 10 times, and all key reactions can be observed in each performance.

## 3. Typical Simulation Results

To explore the oxidative modifications induced by plasma-generated ROS and elucidate the underlying mechanisms of plasma interactions with various organic compounds, this paper compiles and enumerates the oxidative modification products resulting from the interaction of ROS with biological cellular and tissue constituents, as well as environmental organic pollutants. These include intracellular proteins [71,72], DNA [73,74], polysaccharides [75], fatty acids [63], and antibiotics [76,77,78,79,80,81,82,83] and mycotoxins [62,84] (see Table 1 for a detailed list of specific reactants). The impacts of four distinct ROS—OH radicals, O atoms, $O_{3}$ molecules, and $H_{2}O_{2}$ molecules—are analyzed in terms of their reaction types and pathways. These organic substances predominantly consist of C, H, O, and N elements, with certain compounds also containing P, S, Cl, and other elements. Consequently, various reactions involve free ROS interacting with the corresponding elements present in the molecular structures.

### 3.1. H-Abstraction Reactions (Dehydrogenation)

The oxidative modification reactions initiated by ROS typically begin with H-abstraction (dehydrogenation) processes. During these reactions, ROS can abstract H atoms from C, N, O, and S atoms, subsequently combining with them. Specifically, an OH radical often combines with an H atom to form a $H_{2}$O molecule, an O atom combines with an H atom to produce an OH radical, an $O_{3}$ molecule can decompose into an OH radical and an $O_{2}$ molecule following H-abstraction, and an $H_{2}O_{2}$ molecule can decompose into two OH radicals that subsequently participate in H-abstraction reactions. As the most prevalent type of oxidative modification reaction, dehydrogenation can occur at various sites containing H atoms, including methyl (-${\mathrm{CH}}_{3}$), amino (-${\mathrm{NH}}_{2}$), methylene (-${\mathrm{CH}}_{2}$), imino (-NH), thiol (-SH), hydroxyl (-OH), and benzene rings. After an H-abstraction reaction, the initial site becomes metastable and may undergo further oxidative modifications under the influence of ROS.

**Figure 2:**
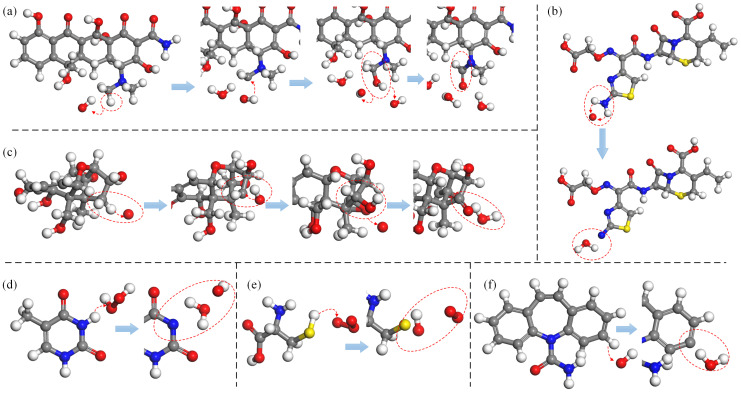
Examples of H-abstraction reactions occurring at various sites in (**a**) tetracycline, (**b**) cefixime, (**c**) deoxynivalenol, (**d**) thymine, (**e**) cysteine, and (**f**) carbamazepine, as previously described in [11,79,85,86,87,88,89,90]. C, N, O, H, S, Cl, and P atoms shown in this and similar figures below are indicated by gray, blue, red, white, yellow, green, and pink spheres, respectively.

Figure 2 presents an illustration of the H-abstraction reaction processes observed in various substances. In Figure 2a, the process is depicted in which an OH radical abstracts the H atom from the terminal methyl group (-${\mathrm{CH}}_{3}$) of the dimethylamino branch (-${\mathrm{NH}\left({\mathrm{CH}}_{3}\right)}_{2}$) in tetracycline (TC), a widely-used antibiotic. Subsequently, another OH radical attaches to the resulting unsaturated site, initiating an addition reaction that introduces hydroxyl groups (-OH). Further dehydrogenation reactions can occur at the newly added OH group and the adjacent C atom, leading to the formation of a double bond at the neighboring unsaturated site and ultimately oxidizing the methyl group (-${\mathrm{CH}}_{3}$) into an aldehyde group (-CHO), which has been experimentally observed as well [79]. Figure 2b illustrates H-abstraction occurring at the amino group (-${\mathrm{NH}}_{2})$ on the aminothiazole ring of cefixime (CFM), resulting in the loss of two H atoms due to the impact of an O atom, which can be witnessed too in [11]. Furthermore, Figure 2c depicts the dehydrogenation reaction of the methylene group (-${\mathrm{CH}}_{2}$) at the C4 position of deoxynivalenol (DON). This reaction can occur under the action of all four types of ROS and is observable in experimental results [85]. Notably, methyl group (-${\mathrm{CH}}_{3}$) dehydrogenation, followed by hydroxylation or ketonization, is specifically observed only in the presence of O atoms and $O_{3}$ molecules. Figure 2d exemplifies the dehydrogenation reaction of the imino group (-NH) in thymine (T), a component of DNA, under the effect of $H_{2}O_{2}$. Furthermore, as depicted in Figure 2e, the thiol group (-SH) in cysteine (Cys) has a high probability of losing an H atom when exposed to all four types of ROS, found observable in the experiment [86,87,88]. Lastly, the benzene ring, as a relatively stable and independent functional group, is still susceptible to C atom dehydrogenation reactions induced by all four types of ROS. Figure 2f provides an example, illustrating the H-abstracton reaction occurring on the benzene ring in carbamazepine (CBZ). In addition, the dehydrogenation reaction can also be found in other studies [89,90].

### 3.2. Bond-Formation Reactions

After ROS abstract H atoms from reaction sites, unpaired free electrons emerge at these locations, allowing them to form electron pairs (bonds) with the free electrons of neighboring atoms. When dehydrogenation occurs at two adjacent atoms, the resulting unsaturated sites can form double bonds. This phenomenon is predominantly observed in simulations as the formation of carbon–carbon (C=C), carbon–nitrogen (C=N), carbon–oxygen (C=O), and nitrogen–oxygen (N=O) double bonds. Since organic reactants primarily consist of carbon, an H-abstraction reaction occurring adjacent to a pre-existing C=C double bond in the molecular structure enables the unsaturated site to establish a double bond with the atom linked through the original double bond, consequently causing the disruption of the initial double bond. Furthermore, when dehydrogenation occurs at the adjacent site on the opposite end of a double bond, the two liberated electrons can pair up, ultimately resulting in the formation of conjugated double bonds (C-C=C-C transforming into C=C-C=C).

**Figure 3:**
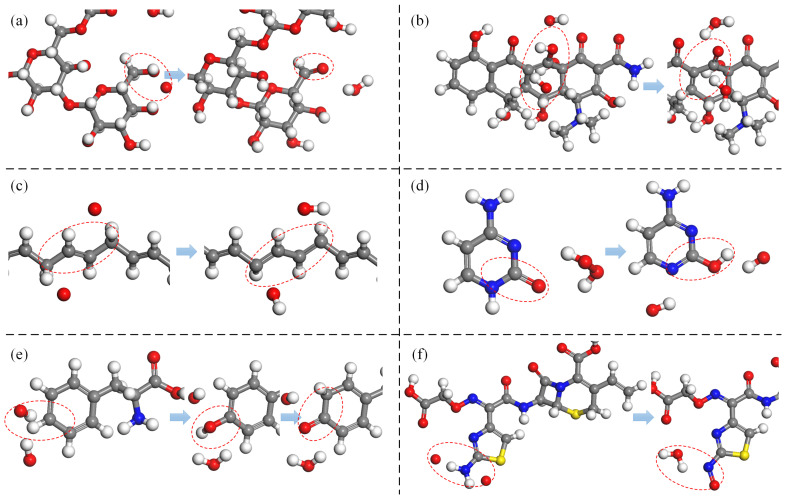
Examples of bond-formation reactions occurring at various sites in (**a**) *Saccharomyces cerevisiae* β-glucan, (**b**) oxytetracycline, (**c**) linolenic acid, (**d**) cytosine, (**e**) phenylalanine, and (**f**) cefixime, as previously described in [11,61,73,78,81,86,88,91].

Figure 3 presents an illustration of double bond formation reactions induced by ROS in various reactants. In Figure 3a, O atoms and OH radicals induce dehydrogenation of a primary alcohol (R-CH_2_OH) within a monosaccharide of *Saccharomyces cerevisiae* β-glucan (SCG). This process results in the formation of a double bond with an adjacent secondary carbon atom. Figure 3b depicts the dehydrogenation of a tertiary carbon atom, shared by adjacent rings in oxytetracycline (OTC), catalyzed by OH radicals. The dehydrogenation leads to the formation of a C=C double bond with a neighboring tertiary carbon atom, causing oversaturation and the subsequent cleavage of the original double bond. Ultimately, a secondary alcohol undergoes dehydrogenation, forming a C=O double bond (ketonization) at the unsaturated site, and experimentally detectable as described [78,81]. Figure 3c illustrates the formation of conjugated double bonds. Under the effect of O atoms, the unsaturated double bonds in linolenic acid undergo H-abstraction from each of the adjacent C atoms flanking the double bond, causing the opening of the double bond and the formation of two new double bonds with adjacent atoms. In chemical structures featuring alternating single and double bonds, this type of reaction may trigger a cascade of single–double bond conversions (bond shifts) within the structure. Figure 3d introduces the formation of a C=N double bond. This occurs between the unsaturated bond site, generated by the reduction of a carbonyl group (C=O) in DNA cytosine (C) under the impact of $H_{2}O_{2}$, and the unsaturated site resulting from the dehydrogenation of an adjacent imino group (-NH) (for details on the reduction reaction, see Section 3.6 below). Ketonization within the phenol structure leads to structural alterations in the benzene ring. As illustrated in Figure 3e, phenylalanine (Phe) undergoes sequential dehydrogenation and hydroxyl addition reactions in the presence of O and OH radicals, ultimately forming phenol [86]. The secondary alcohol can be further oxidized to a ketone group (C=O), and the double bond within the benzene ring is converted into a single bond [88]. The formation of N=O double bonds typically occurs through the direct addition of an O atom following a dehydrogenation reaction on an amino group (-${\mathrm{NH}}_{2}$). An example of this is the oxidative modification of the amino group (-${\mathrm{NH}}_{2}$) in the aminothiazole ring of cefixime [11], as depicted in Figure 3f. In addition, the bond-formation reaction can also be found in other studies [61,73,91].

### 3.3. Oxygen-Addition Reactions

After the dehydrogenation reaction, the unsaturated sites exhibit the capability not only to form double bonds with neighboring sites but also to engage in O-addition reactions with ROS, ultimately achieving saturation. O-addition reactions can be considered a subset of bonding reactions, primarily involving the formation of alcohol groups (-OH) through OH addition and the direct incorporation of O atoms. These addition reactions predominantly occur on C and N elements. Following the addition of OH radicals, which leads to the formation of alcohol groups (-OH), these groups can undergo further oxidation in the presence of ROS, resulting in the formation of aldehyde (-CHO), ketone (-C=O), or carbonyl (R-C=O) groups. Under high ROS concentrations, the formation of carboxyl groups (-COOH) may also be observed. The direct addition of O atoms requires the abstraction of two or more H atoms at the reaction site and is frequently observed in reactions where O atoms act as ROS.

**Figure 4:**
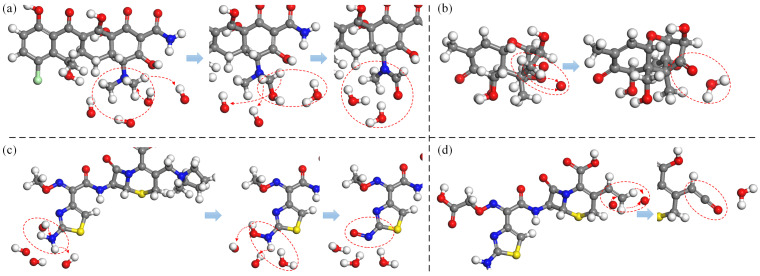
Examples of O-addition reactions occurring at various sites in (**a**) chlortetracycline, (**b**) deoxynivalenol, (**c**) cefepime, and (**d**) cefixime, as previously described in [61,79,80,86,89].

Figure 4 presents an illustration of O-addition reactions for various substances. Figure 4a depicts the reaction of oxygen addition to the dimethylamino group (-${\mathrm{NH}\left({\mathrm{CH}}_{3}\right)}_{2}$) of chlortetracycline (CTC), catalyzed by OH radicals. Similar to TC, the reaction predominantly involves dehydrogenation and OH addition, leading to structural transformations from methyl (-${\mathrm{CH}}_{3}$) to methanol (-${\mathrm{CH}}_{2}\mathrm{OH}$) and subsequently to formaldehyde (-CHO), appearing in the experiment as well [79,80]. A comparable process occurs in methylene groups (-${\mathrm{CH}}_{2}$), ultimately resulting in the formation of ketone or carbonyl (-C=O) structures, as exemplified in Figure 4b, which illustrates the reaction of methylene (-${\mathrm{CH}}_{2}$) in DON upon the impact of O and $O_{3}$. Notably, the reaction chains -${\mathrm{CH}}_{3}$ → -${\mathrm{CH}}_{2}\mathrm{OH}$ → -CHO and -${\mathrm{CH}}_{2}$ → -CHOH → -C=O are not exclusive to methyl (-${\mathrm{CH}}_{3}$) and methylene (-${\mathrm{CH}}_{2}$) groups; structures that inherently contain alcohol groups (-OH) can also undergo subsequent reactions, leading to the formation of aldehyde (-CHO) or carbonyl (-C=O) groups. Figure 4c illustrates the O-addition reaction at the amino group (-${\mathrm{NH}}_{2}$) within the aminothiazole ring of cefepime (FEP). Under the effect of OH radicals, the oxidation process resembles cascade oxidation, where OH addition is followed by dehydrogenation, leading to the formation of a double bond. In the presence of O atoms and $O_{3}$ molecules, the amino group (-${\mathrm{NH}}_{2}$) undergoes the loss of two H atoms, followed by the direct addition of an O atom, resulting in the formation of an N=O double bond, which represents the primary formation mechanism. Similarly, C atoms can undergo direct O addition to form C=O double bonds. As depicted in Figure 4d, the vinyl terminal (-C=C) undergoes dehydrogenation in the presence of O atoms, followed by the direct addition of an O atom to form a C=C=O structure. Under the impact of $O_{3}$, the formation of this structure involves an intermediary step in which an alcohol group (-OH) is formed first. In addition, the O-addition reaction can also be found in other studies [61,86,89,92,93].

### 3.4. Bond-Breakage Reactions

In addition to the bonding reactions described in the preceding sections, oxidative modifications mediated by ROS can also trigger bond-cleavage reactions, similar to dehydrogenation (i.e., R-H bond dissociation). These bond-cleavage reactions are generally initiated either by the direct intervention of ROS or by the oversaturation of bonding sites. Such cleavages predominantly occur at C-C, C-N, C-O, and N-O bonds. However, the specific positioning of these bonds within the molecular structure can lead to diverse oxidative modification outcomes, including functional group loss, side-chain decomposition, primary structure cleavage, and ring-opening reactions.

**Figure 5:**
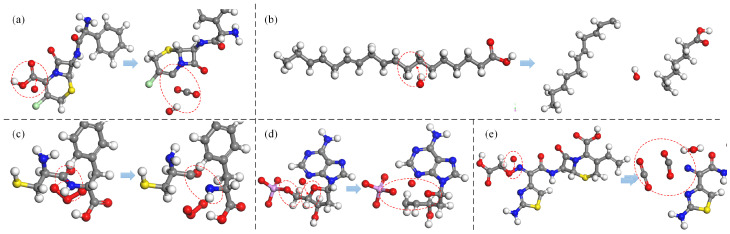
Examples of bond-breakage reactions occurring at various sites in (**a**) cefaclor, (**b**) linolenic acid, (**c**) polypeptides, (**d**) deoxyribose, and (**e**) cefixime, as previously described in [61,86,89,91,94,95,96,97,98].

Figure 5 presents an illustration of the various oxidative modifications resulting from bond-cleavage reactions. Figure 5a depicts a decarboxylation reaction, in which the C-C bond within a carboxyl group (-COOH) undergoes cleavage under the impact of ROS, resulting in the detachment of the carboxyl group (-COOH) from the parent molecule. The detached fragment is subsequently oxidized to form ${\mathrm{CO}}_{2}$ molecules, a phenomenon observed in amino acids, cephalosporin antibiotics, fatty acids, and other compounds [94,95]. In unsaturated fatty acids, such as linolenic acid, C-C bond cleavage induced by ROS can also lead to the disruption of the main structural framework, as illustrated in Figure 5b. The peptide bond, which is the most prevalent C-N bond in proteins and links two amino acids, is the focus of Figure 5c. This subfigure illustrates the cleavage of peptide bonds by ROS, leading to the disruption of polypeptide and protein structures. C-O bonds are widespread in various organic compounds, and Figure 5d provides an example using deoxyribose in DNA. Here, C-O bond cleavage within the deoxyribose structure can directly result in the detachment of phosphate groups or indirectly cause the loss of nitrogenous bases, thereby compromising the DNA chain structure. Finally, the N-O bond is particularly susceptible to cleavage [95], as demonstrated in Figure 5e. This subfigure presents the detachment of the carboxymethoxyimino group in CFM, where the cleavage rate of the N-O bond approaches almost 100% under the influence of all four types of ROS. In addition, the bond-breakage reaction can also be found in other studies [61,86,89,91,96,97,98].

### 3.5. Reaction with Common Functional Group

Various functional groups exist in the organic compounds that have been statistically analyzed. Due to their consistent structures and specific chemical properties, the types of oxidative modifications induced by ROS exhibit certain similarities. Common functional groups that interact with ROS include hydroxyl (-OH), carboxyl (-COOH), aldehyde (-CHO), carbonyl (-C=O), amino (-${\mathrm{NH}}_{2}$), and chloro (-Cl) groups. Additionally, reactions occurring on identical structures often display similar trends, such as those involving double bonds, benzene rings, ether bonds, and epoxide rings.

**Figure 6:**
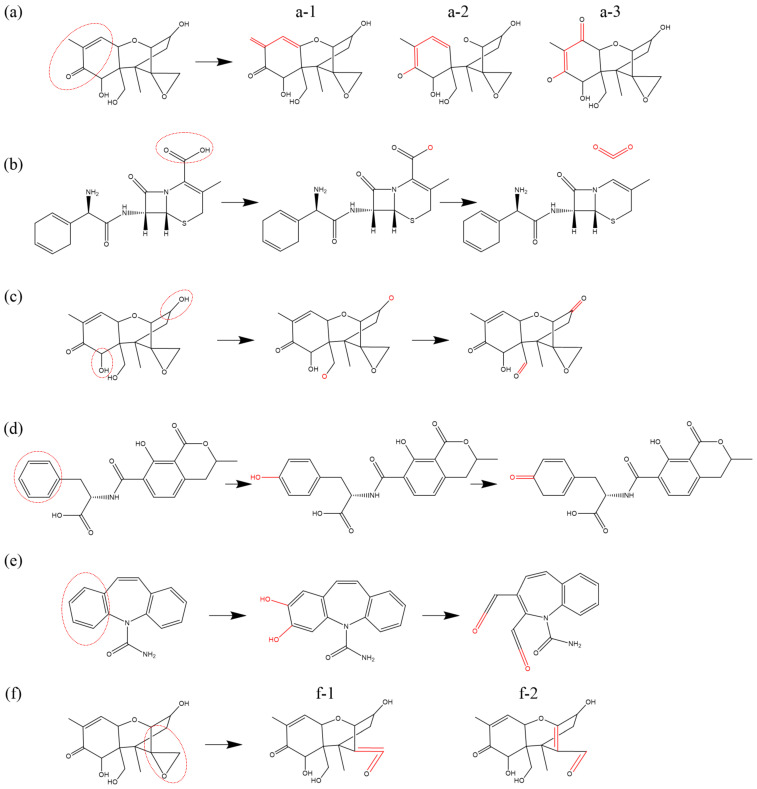
Oxidative modification pathways induced by ROS: (**a**) disruption of double bonds in deoxynivalenol via dehydrogenation and the formation of three conjugated double bonds is shown in subfigures (**a-1**), (**a-2**), and (**a-3**), respectively. (**b**) ROS-induced decarboxylation reaction in cefradine, (**c**) transformation of hydroxyl (-OH) to ketone (-C=O) via ketonization in deoxynivalenol, (**d**) oxidation of benzene rings in ochratoxin B leading to ring destabilization, (**e**) oxygen-addition reaction in carbamazepine, and (**f**) ring-opening of epoxide in deoxynivalenol, and two different oxidative modifications are shown in subfigures (**f-1**) and (**f-2**), respectively, as previously described in [78,83,89,91,92,93,95,97,99]. For clarity, H atoms bound to the C atoms are not shown.

Figure 6 provides a comprehensive overview of the oxidative modification reactions affecting these functional groups and analogous structures. Figure 6a illustrates the reductive destruction of toxic double bonds in DON. The formation of conjugated double bonds via dehydrogenation reactions, the transition from single to double bonds due to C-O bond cleavage, and the direct addition of OH radicals can either indirectly or directly contribute to the disruption of double bonds [99]. Figure 6b presents the decarboxylation reactions of carboxyl groups (-COOH) in various substances under the influence of ROS [95], whereas Figure 6c depicts the oxidation process, where hydroxyl groups (-OH) are converted into aldehyde (-CHO) or carbonyl (-C=O) groups [92,93,97]. Amino (-${\mathrm{NH}}_{2}$) and chloro (-Cl) groups primarily undergo dehydrogenation reactions and bond cleavage [78,83], leading to their detachment under the impact of ROS. As shown in Figure 6d, the oxidative modifications of benzene rings in the presence of OH radicals and $H_{2}O_{2}$ include dehydrogenation reactions, OH-addition leading to phenol formation, and the oxidation of phenols, where the hydroxyl group (-OH) is transformed into a carbonyl (-C=O) group. With an increase in ROS dosage, benzene rings may also form catechol or hydroquinone. At higher $O_{3}$ concentrations, superoxide addition reactions can occur, resulting in the formation of a diketone structure following C-C bond cleavage, as demonstrated in Figure 6e. Ether bonds (C-O-C), consisting of two C-O single bonds, are highly susceptible to cleavage under the influence of ROS, particularly in the disruption of glycosidic bonds. Notably, when ether bonds adopt an epoxide ring structure, they can undergo ring-opening reactions in the presence of ROS. The unsaturated sites generated after bond cleavage can continue to undergo oxidation, leading to the formation of double bonds, carbonyl (-C=O), or aldehyde (-CHO) structures. The detailed reaction processes are outlined in Figure 6f. In addition, the oxidative modification reactions of of other functional groups can also be found in other studies [89,91,100,101,102].

### 3.6. Reactions with Specific ROS

**Figure 7:**
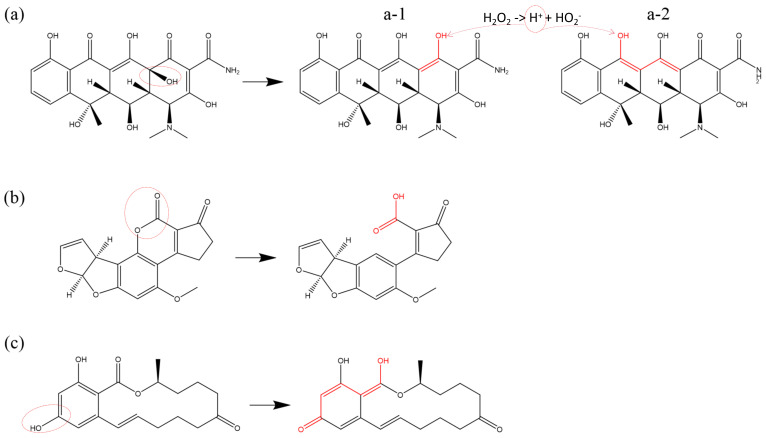
Oxidative modification pathways induced by $H_{2}O_{2}$: (**a**) dehydroxylation in oxytetracycline and transformation of ketone (-C=O) to hydroxyl (-OH) via bond shifts (the hydrogen atoms abstracted from the decomposition of $H_{2}O_{2}$); two different oxidative modification pathways are shown in subfigures (**a-1**) and (**a-2**), respectively. (**b**) Transformation of ketone (-C=O) to hydroxyl (-OH) in aflatoxin $B_{1}$ leading to ring opening and (**c**) ROS-induced oxidation of hydroxyl (-OH) on benzene ring in zearalenone leading to the transformation of ester group (-C=O) of the lactone ring to hydroxyl (-OH) via bond shifts, as previously described in [11,80,96].

While the oxidation reactions previously discussed predominate in the interactions between the four types of ROS (i.e., OH radicals, O atoms, $O_{3}$ molecules, and $H_{2}O_{2}$ molecules) and diverse organic compounds, $H_{2}O_{2}$ molecules exhibit an additional oxidative modification mechanism: the decomposition of $H_{2}O_{2}$ into H atoms and ${\mathrm{HO}}_{2}$ radicals, which exerts a reducing effect on reactants. Specific reaction types include OH-abstraction (dehydroxylation) reactions and H-addition reactions. Typically, hydroxyl groups (-OH) undergo oxidation to form C=O double bonds under the impact of O atoms, OH radicals, and $O_{3}$ molecules. However, $H_{2}O_{2}$ molecules have the capacity to remove a hydroxyl group (-OH) from the tertiary alcohol in oxytetracycline (OTC). Subsequently, the unsaturated bond site resulting from dehydroxylation can form a double bond with adjacent atoms [11,80], thereby achieving saturation (as illustrated in Figure 7a). Dehydroxylation reactions are also observed in SCG and DON. The H-addition reaction primarily targets C=O double bonds, reducing carbonyl or ketone groups (-C=O) to hydroxyl groups (-OH). This reaction can proceed through both direct and indirectly induced mechanisms. For instance, in Figure 7b, the O atom of C=O carbonyl on the lactone ring in aflatoxin $B_{1}$ ($\mathrm{AF}B_{1}$) undergoes direct H-addition reaction under the impact of $H_{2}O_{2}$. This is followed by the formation of a double bond between the unsaturated site and an O atom within the ring, ultimately leading to the ring-opening of the lactone ring [96]. In the case of zearalenone (ZEN), the carbonyl group (-C=O) can be reduced through an indirectly induced mechanism. Here, the para-hydroxyl group on the benzene ring is oxidized to form a ketone (-C=O) group. Oversaturation then leads to bond shifts and transfers to C=O in the ester (-COOR) group, causing it to be reduced to a single bond and forming an OH group with a free H atom, as depicted in Figure 7c.

In addition, under the action of $O_{3}$ molecules, superoxide addition to the benzene ring results in the disruption of the benzene ring structure and the formation of a diketone structure, which can also be regarded as an oxidative modification induced by specific ROS.

### 3.7. Reactions with Specific Element

Among the diverse array of organic compounds analyzed, the oxidative modification reactions previously described predominantly involve the elements carbon (C), nitrogen (N), oxygen (O), and hydrogen (H). Additionally, sulfur (S)—found in methionine (Met) and cysteine (Cys) in amino acids, as well as in cephalosporin antibiotics—and chlorine (Cl)—present in tetracycline antibiotics—can also interact with ROS. Notably, although the phosphate group in DNA contains phosphorus (P), ROS do not directly interact with P.

**Figure 8:**
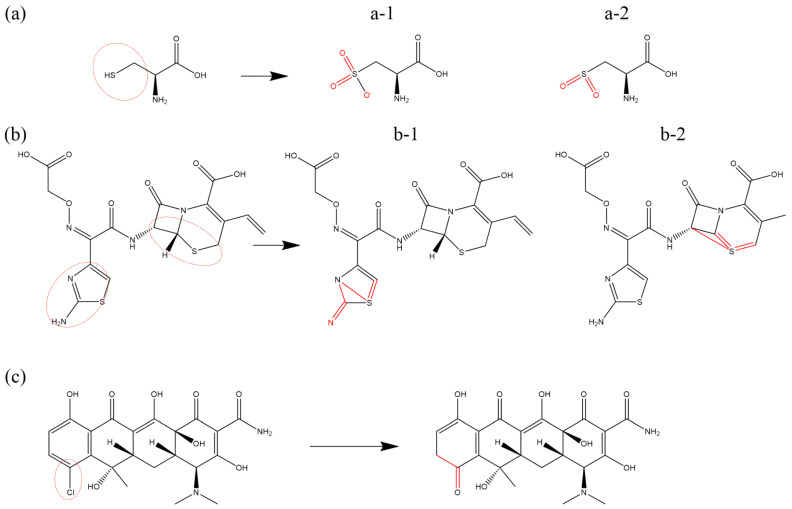
Oxidative modification pathways of sulfur (S) and chlorine (Cl) induced by ROS: (**a**) sulfonation of thiol group (-SH) in cysteine after dehydrogenation; two different oxidative modifications are shown in subfigures (**a-1**) and (**a-2**), respectively. (**b**) Bond formations on S atoms of aminothiazole ring and the six-membered thiazine ring in cefixime are shown in subfigures (**b-1**) and (**b-2**), respectively, and (**c**) ROS-induced detachment of Cl atoms in chlortetracycline leading to ring destabilization, as previously described in [83,86,87,88,103].

Beyond the dehydrogenation of the thiol group (-SH) in Cys previously mentioned, a common reaction involving sulfur (S) is the addition of O atoms [86,87,88,103]. Figure 8a illustrates the formation of a sulfonate group in Cys through the incorporation of three oxygen atoms under the impact of OH radicals. When only two oxygen atoms are added, the resultant structure is sulfinic acid (Figure 8b). Similar oxygen-addition reactions are also observed in CFM. Moreover, both the aminothiazole ring and the six-membered thiazine ring in CFM contain S elements, which can form double bonds with adjacent sites or single bonds with nearby atoms under the impact of ROS. The oxidized products are illustrated in Figure 8c

Chlorine (Cl) is a constituent of chlortetracycline (CTC) and demeclocycline (DMC). Under the influence of ROS, bond cleavage occurs in the chloro (-Cl) group, resulting in the detachment of chlorine. The unsaturated bond site exposed after cleavage can then add an OH radical and undergo ketonization, forming a C=O double bond [83]. Figure 8c depicts the corresponding reaction process.

### 3.8. Chain Structures

Having detailed the diverse fundamental types of oxidative modification reactions in preceding sections, this article now expands on the intricate interactions between ROS and organic compounds. These interactions go beyond specific dehydrogenation, addition, bond-cleavage, or bond-formation reactions; instead, they represent a complex interplay of various reaction types, ultimately yielding intricate final products. The oxidative modification outcomes vary significantly depending on the location and structural differences of the reaction sites. Consequently, this article further classifies organic compounds, based on their primary structures, into two categories: those predominantly featuring chain structures and those primarily composed of ring structures. It then delves into the oxidative modification results stemming from the combination of multiple fundamental reaction types in these reactants.

**Figure 9:**
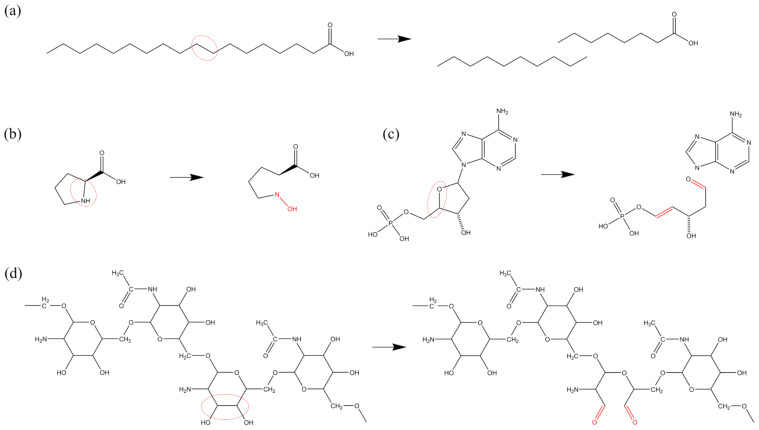
Oxidative modification pathways of chain structures induced by ROS: (**a**) bond dissociation in stearic acid, (**b**) ring opening in proline, (**c**) bond breakage in deoxyribose leading to ring opening, and (**d**) transformation of hydroxyl (-OH) to aldehyde (-C=O) in Poly-β-1-6-N-acetylglucosamine leading to ring opening.

In this article, the term “chain structures” encompasses not only substances with a singular long-chain molecular configuration, such as fatty acids, but also those composed of repetitive monomeric units linked in a chain-like fashion through specific bonds, including proteins, DNA, and polysaccharides. Fatty acids, characterized by their relatively small molecular size and a single-chain structure, predominantly undergo basic oxidative modifications, including the formation of double bonds and conjugated double bonds, cascade oxidation of methyl (-${\mathrm{CH}}_{3}$) and methylene (-${\mathrm{CH}}_{2}$) groups, and the detachment of carboxyl (-COOH) groups. The cleavage of C-C single bonds signifies the disruption of the molecular architecture, as illustrated in Figure 9a, where stearic acid undergoes bond cleavage in the presence of high-concentration $O_{3}$ molecules, resulting in the fragmentation of its molecular structure.

Proteins, composed of amino acids linked by peptide bonds, primarily experience oxidative modifications in their side chains and peptide bonds. These modifications mainly involve dehydrogenation reactions and O-addition reactions, while high concentrations of ROS can also induce peptide bond cleavage, thereby compromising the protein’s structural integrity. Figure 9b depicts the cleavage of the five-membered ring in the proline (Pro) side chain by OH radicals and O atoms, leading to ring opening.

DNA forms a chain through phosphodiester bonds and adopts a double-helix structure through hydrogen bonding between nitrogenous bases. ROS can disrupt the C-O bond in phosphodiester bonds, causing DNA cleavage. Its monomeric constituent, deoxyribonucleotide, features a ring structure, and the oxidative modifications of nitrogenous bases by ROS primarily encompass dehydrogenation reactions, bond-formation reactions, and O-addition reactions. Figure 9c illustrates the C-O bond cleavage caused by deoxyribose dehydrogenase, resulting in the separation of the nitrogenous base from the pentose sugar.

Polysaccharide structures are composed of monosaccharides linked by glycosidic bonds. The oxidative modifications of the two polysaccharides examined in this article, SCG and Poly-β-1-6-N-acetylglucosamine (PNAG), by ROS primarily focus on the monosaccharide molecules and glycosidic bonds. Within monosaccharides, the reactions mainly involve dehydrogenation reactions, bond-formation reactions, and O-addition reactions, while at glycosidic bonds, the primary reactions are bond-cleavage and subsequent O-addition reactions. Figure 9d presents the dehydrogenation of adjacent hydroxyl (-OH) groups in the monosaccharide structure of PNAG by O atoms, followed by C-C bond cleavage and ultimately O addition to form a dialdehyde structure.

### 3.9. Ring Structures

In this article, ring structures predominantly appear as individual molecular entities containing various cyclic moieties, such as cephalosporin antibiotics, tetracycline antibiotics, carbamazepine, and mycotoxins. The oxidative modifications of these ring structures primarily involve dehydrogenation reactions, bond-formation reactions, O-addition reactions, and bond cleavage reactions. However, the presence of multiple rings within these molecules introduces additional reaction pathways, including ring opening and ring formation, resulting in more intricate oxidative modification outcomes.

**Figure 10:**
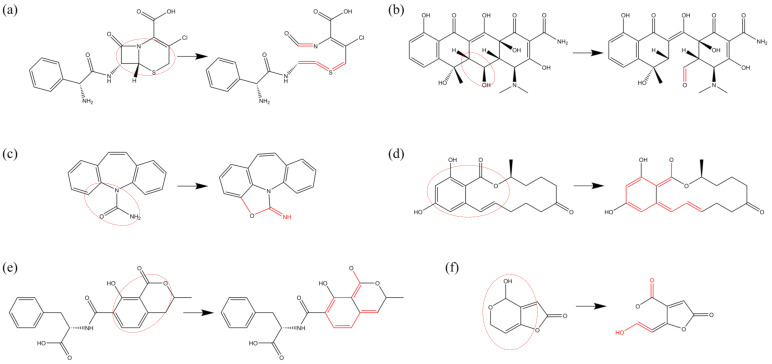
Oxidative modification pathways of ring structures induced by ROS: (**a**) bond breakage and formation of β-lactam ring in cefaclor, (**b**) transformation of hydroxyl (-OH) to aldehyde (-C=O) in tetracycline leading to ring opening, (**c**) disruption of double bonds of amide group in carbamazepine via dehydrogenation leading to ring formation, (**d**) dehydrogenation in zearalenone and disruption of ketone (-C=O) via bond shifts, (**e**) dehydrogenation in ochratoxin B and disruption of ketone (-C=O) via bond shifts, and (**f**) ROS-induced cleavage of ether linkages in patulin leading to ring opening.

For cephalosporins, the reaction sites are distributed across various functional groups and within the rings themselves, with all four fundamental reaction types occurring under ROS influence. Owing to the proximity of the β-lactam ring and the six-membered thiazine ring in their molecular structure, C-C and C-N bond cleavages can occur under ROS action, leading to the opening of both rings. The unsaturated bond positions subsequently form bonds with adjacent atoms, as illustrated in Figure 10a.

The oxidative modifications of tetracycline antibiotics are primarily concentrated on the functional groups of the side chains attached to their rings, with all four fundamental reaction types directly observable on these side chains. Figure 10b depicts the dehydrogenation of the OH group on the third six-membered ring (from the left) of oxytetracycline (OTC) under $O_{3}$ action, followed by oxidation to a C=O bond. The oversaturation of the secondary carbon atom results in C-C bond cleavage, leading to the opening of the six-membered ring.

Figure 10c illustrates the dehydrogenation of carbamazepine under $O_{3}$ action, forming unsaturated bond positions that subsequently interact with the structurally altered amide group, leading to the formation of a five-membered heterocyclic ring.

Mycotoxins, particularly those belonging to the lactone ring class, invariably contain lactone rings. The structure of ZEN, for instance, includes a benzene ring and a 14-membered macrolide lactone ring, with multiple double bonds within the inner ring. Under ROS influence, dehydrogenation occurs at the C position, forming a double bond with the adjacent carbon atom. Following the principle of conjugated double-bond formation, a cascade of oxidation reactions ensues, leading to single- and double-bond shifts within the ring, ultimately resulting in the reduction of the C=O bond on the opposite side of the ring. This reaction process is illustrated in Figure 10d.

Similar oxidative modifications occur in ochratoxin B (OTB). Figure 10e presents the oxidative modification in OTB, where carbon undergoes dehydrogenation, forming a double bond and leading to the reduction of the C=O bond. Alterations in bond positions within the ring result in significant transformations in the molecular structure.

In patulin (PAT), the cleavage of the C-O bond in the hemiacetal structure directly induces a ring-opening reaction. The primary types of oxidative modifications remain O-addition reactions, dehydrogenation reactions, and bond-cleavage reactions. The specific reaction process is illustrated in Figure 10f.

### 3.10. Summary

According to the reactive MD simulation results, a variety of reactions resulting from oxidative modifications induced by ROS were systematically classified and illustrated through examples involving several organic compounds, and thus a concise overview of the simplified reaction sites and the corresponding reaction types can be provided, which is presented in Table 2.

Typically, ROS-induced oxidative modifications initiate with dehydrogenation reactions, which result in the formation of unsaturated bond positions. These newly formed unsaturated bonds are highly susceptible to bond formation, addition reactions, and other subsequent transformations. When adjacent C atoms undergo dehydrogenation, double bonds are formed. Conversely, dehydrogenation of atoms neighboring existing double bonds leads to the creation of conjugated double bonds, a reaction pattern that can propagate through a series of oxidative cascades, resulting in shifts between single- and double-bond positions. Following dehydrogenation, methyl (or methylene) groups can undergo hydroxylation, forming alcohol groups, which may subsequently be oxidized further to aldehyde (or carbonyl) groups. Alcohol groups already present in the molecule can also undergo oxidation to carbonyl groups, and bond position shifts may occur due to oversaturation at the initial site after bond formation. H-abstraction and dehydroxylation reactions can transpire under the influence of $H_{2}O_{2}$. Carboxyl groups, when exposed to ROS, can undergo dehydrogenation or direct detachment (decarboxylation), leading to the formation of ${\mathrm{CO}}_{2}$ molecules. C-C bond cleavage reactions can result in molecular fragmentation, the detachment of functional groups, and the formation of hydroxyl, ketone, and carboxyl groups at the newly formed unsaturated bond positions. Benzene rings, under the action of ROS, undergo dehydrogenation and hydroxyl addition reactions. High concentrations of ROS can also induce OH oxidation, leading to the formation of C=O bonds and shifts in bond positions, thereby altering the fundamental structure of the benzene ring. In the presence of $O_{3}$ molecules, benzene rings can undergo superoxide addition, ultimately resulting in ring cleavage. Furthermore, C=O bonds can be reduced to OH groups under the impact of $H_{2}O_{2}$, and unsaturated positions can form bonds with adjacent atoms. C=C bonds may be directly cleaved into single bonds through hydroxylation or undergo indirect changes in bond type due to reactions occurring at other positions within the molecule. C-O bonds, which are present in ether and ester linkages, are particularly prone to cleavage when exposed to ROS, leading to oxidation modifications such as molecular fragmentation and ring opening.

The fundamental reaction types involving N atoms share similarities with those of C atoms. They primarily include dehydrogenation, the formation of C=N double bonds with C atoms, the addition of OH groups, oxidation to N=O, or direct addition of O atoms (a process known as nitrosylation). These oxidative modifications may also involve the disruption of molecular structures and the detachment of functional groups due to N-C and N-O bond cleavages. The oxidative modifications of S atoms are mainly characterized by dehydrogenation reactions, O-addition reactions, and bond-formation reactions. Dehydrogenation is specific to thiol groups, whereas S atoms can undergo oxidation by adding O atoms to form sulfonic or sulfinic acid structures (a process termed sulfonation). When S atoms are within rings or surrounded by other atoms, bond-formation reactions may also take place. As for Cl atoms, their fundamental reaction type under the influence of ROS exclusively involves the detachment of chlorine groups due to bond cleavage reactions. The unsaturated sites that emerge following bond cleavage can subsequently undergo OH addition and oxidation to form C=O bonds.

Reactive MD simulations have significantly advanced our understanding of chemical reactions at the molecular level. However, they are not without limitations. One major constraint lies in modeling ROS interactions at longer time scales typically beyond nanoseconds. Reactive MD simulations typically struggle to capture the slow, long-term processes that ROS are involved in, such as their cumulative effects on biological molecules over nanoseconds. This is because the computational cost of running simulations for extended time frames becomes prohibitively high. Moreover, when it comes to larger biological systems, reactive MD faces challenges. Biological systems are extremely complex, with a vast number of atoms and intricate interactions. Representing all these details accurately in one simulation is difficult. For example, in a large-scale protein–ROS interaction scenario, the simulation may not be able to account for all the conformational changes and intermolecular forces effectively due to the limitations in computational resources. To overcome these limitations, future directions are being explored. Incorporating hybrid quantum mechanics/molecular mechanics (QM/MM) methods shows great promise [104]. QM can accurately describe the electronic structure and chemical reactions, while MM can handle the large-scale molecular motions efficiently. RMD simulations, typically implemented using reactive force fields such as ReaxFF, offer a unique balance between system size and chemical fidelity, enabling the study of explicit bond-breaking and -formation processes in systems containing thousands to millions of atoms. This makes RMD particularly suitable for investigating complex reaction networks under nitro-oxidative stress conditions. In contrast, traditional MD simulations, while capable of handling similarly large systems and longer timescales (nanoseconds to microseconds), lack the ability to model chemical reactions due to their reliance on fixed-topology force fields. On the other hand, QM/MM methods provide higher accuracy for reaction modeling by treating the reactive region quantum mechanically, but they are limited to much smaller systems (hundreds to thousands of atoms) and shorter timescales (femtoseconds to picoseconds) due to their substantial computational cost. By combining these two methods, we can achieve better accuracy in simulating plasma-generated ROS interactions. For instance, in studying the reaction of ROS with a biomolecule, QM can be used to model the reactive sites precisely, while MM can manage the rest of the large-scale molecular environment. This approach allows for a more comprehensive and accurate understanding of the complex processes involving ROS in biological systems, opening new doors for research in fields like medicine and environmental science.

## 4. Conclusions

This review paper systematically investigates the oxidative modification mechanisms of biological tissue components and environmental toxins induced by ROS generated from CAP, examining the microscopic processes of these complex reactions through reactive MD simulations. The results reveal that ROS primarily oxidize and modify organic compounds through reaction types such as dehydrogenation, bond formation, oxygen addition, and bond cleavage, yielding a variety of products. These reactions are not only influenced by the type and concentration of ROS but are also closely related to the molecular structure of the reactants. For instance, chain-structured compounds, such as fatty acids and proteins, primarily undergo bond cleavage and side-chain oxidation, whereas ring-structured compounds, such as antibiotics and mycotoxins, exhibit more complex reaction pathways, including ring opening/closing and bond rearrangement. Additionally, specific ROS, such as $H_{2}O_{2}$, exhibit unique reductive effects. This paper provides an in-depth theoretical framework for understanding the molecular mechanisms of CAP-induced oxidative modifications, which is essential for optimizing plasma-based biomedical therapies, sterilization techniques, and environmental decontamination strategies. In future study, given that the RONS in CAP are primarily small molecular species with limited types, it would be feasible to construct a specialized reactive force field tailored for plasma medicine studies by machine learning algorithms, which could potentially further optimize existing reactive MD simulations in plasma medicine applications, enriching the database of oxidative modification reactions, and promote the application of CAP technology across a wider spectrum of fields.

## Figures and Tables

**Table 1:** Classification of organic reactants analyzed in this study.

Category	Reactants
Proteins	Amino Acids (20), Polypeptides (2)
DNA	Nitrogenous Bases (4), Deoxyribose, DNA Strands
Carbohydrates	*Saccharomyces cerevisiae* β-glucan (SCG), Poly-β-1-6-N-acetylglucosamine (PNAG)
Fatty Acids	Oleic Acid, Linoleic Acid, Linolenic Acid, Stearic Acid, Palmitic acid
Tetracyclines	Tetracycline (TC), Oxytetracycline (OTC), Chlortetracycline (CTC), Demeclocycline (DMC)
Cephalosporins	Cefradine (CRD), Cefaclor (CEC), Cefixime (CFM), Cefepime (FEP)
Drugs	Carbamazepine (CBZ)
Mycotoxins	Deoxynivalenol (DON), Aflatoxin $B_{1}$ (AF$B_{1}$), Zearalenone (ZEN), Ochratoxin B (OTB), Patulin (PAT)

**Table 2:** Fundamental oxidative modification reactions and their products.

Elements	Structure	Oxidative Modification Products
Carbon (C)	C-H	C-H (dehydrogenation)
	C-C	C=C (double-bond formation)
	C-C=C-C	C=C-C=C (conjugated double-bond formation)
	-${\mathrm{CH}}_{3}$	-${\mathrm{CH}}_{2}\mathrm{OH}$/-CHO (hydroxylation → aldehyde formation)
	-${\mathrm{CH}}_{2}$-	-CHOH/-C=O (hydroxylation → ketonization)
	-C-OH	C=O (double-bond formation via C=C shifting)
		-OH release ($H_{2}O_{2}$-induced oxidation)
	-C(=O)NH	-C-O/C=N (dehydrogenation and oxidation, leading to bond shifts)
		-C-OH ($H_{2}O_{2}$-induced oxidation)
	-COOH	Decarboxylation → ${\mathrm{CO}}_{2}$ release
	C-C breakage	-OH/=O/-OOH (ROS-induced cleavage)
	benzene-H	-OH/=O (hydroxylation and oxidation, leading to C=C shifts)
		Ring opening ($O_{3}$-induced oxidation)
	C=C	C-C (hydroxylation-induced saturation)
	C-O (C-O-C)	Bond breakage (ROS-induced cleavage of ether linkages)
Nitrogen (N)	N-H	N-H (dehydrogenation)
	N-C	N=C (double-bond formation)
	-${\mathrm{NH}}_{2}$	-NHOH/-N=O (oxidation)
	-NH-	-NOH/-N=O (limited occurrence)
	N-C breakage	Ring opening (via ROS cleavage)
	N-O breakage	Bond cleavage
Sulfur (S)	S-H	S-H (dehydrogenation)
	-S-	-${\mathrm{SO}}_{3}$/-${\mathrm{SO}}_{2}$(sulfonation)
	S-C	S=C (double-bond formation)
	S,C,N interactions	S-C, S-N bond formation
Chlorine (Cl)	-Cl	Chlorine detachment (-Cl loss)

## Data Availability

The data that support the findings of this study are available from the corresponding author upon reasonable request, and the Smiles of modification of organic compounds collected in this study can be accessed via https://github.com/SDU-HV-Plasma/Smilesoforganiccompounds, accessed on 23 April 2025.

## References

[1] Kong M.G., Kroesen G., Morfill G., Nosenko T., Shimizu T., Van Dijk J., Zimmermann J. (2009). Plasma medicine: An introductory review. New J. Phys..

[2] Neyts E.C., Yusupov M., Verlackt C.C., Bogaerts A. (2014). Computer simulations of plasma–biomolecule and plasma–tissue interactions for a better insight in plasma medicine. J. Phys. D Appl. Phys..

[3] Dai Dong N.W., Tao S. (2017). A review on the state of art and future trends of atmospheric pressure low temperature plasmas. Trans. China Electrotech. Soc..

[4] Laroussi M., Bekeschus S., Keidar M., Bogaerts A., Fridman A., Lu X., Ostrikov K., Hori M., Stapelmann K., Miller V. (2021). Low-temperature plasma for biology, hygiene, and medicine: Perspective and roadmap. IEEE Trans. Radiat. Plasma Med. Sci..

[5] Deilmann M., Halfmann H., Bibinov N., Wunderlich J., Awakowicz P. (2008). Low-pressure microwave plasma sterilization of polyethylene terephthalate bottles. J. Food Prot..

[6] Moreau M., Orange N., Feuilloley M. (2008). Non-thermal plasma technologies: New tools for bio-decontamination. Biotechnol. Adv..

[7] Morrison C.F. (1977). Electrosurgical Method and Apparatus for Initiating an Electrical Discharge in an Inert Gas Flow. US Patent.

[8] Fridman G., Friedman G., Gutsol A., Shekhter A.B., Vasilets V.N., Fridman A. (2008). Applied plasma medicine. Plasma Process. Polym..

[9] Farin G., Grund K. (1994). Technology of argon plasma coagulation with particular regard to endoscopic applications. Endosc. Surg. Allied Technol..

[10] Selcuk M., Oksuz L., Basaran P. (2008). Decontamination of grains and legumes infected with Aspergillus spp. and Penicillum spp. by cold plasma treatment. Bioresour. Technol..

[11] Zhang T., Zhou R., Wang P., Mai-Prochnow A., McConchie R., Li W., Zhou R., Thompson E.W., Ostrikov K.K., Cullen P.J. (2021). Degradation of cefixime antibiotic in water by atmospheric plasma bubbles: Performance, degradation pathways and toxicity evaluation. Chem. Eng. J..

[12] Bárdos L., Baránková H. (2010). Cold atmospheric plasma: Sources, processes, and applications. Thin Solid Film..

[13] Sharma A., Pruden A., Yu Z., Collins G.J. (2005). Bacterial inactivation in open air by the afterglow plume emitted from a grounded hollow slot electrode. Environ. Sci. Technol..

[14] Sladek R., Stoffels E. (2005). Deactivation of *Escherichia coli* by the plasma needle. J. Phys. D Appl. Phys..

[15] Shekhter A.B., Serezhenkov V.A., Rudenko T.G., Pekshev A.V., Vanin A.F. (2005). Beneficial effect of gaseous nitric oxide on the healing of skin wounds. Nitric Oxide.

[16] Davydov A., Kuchukhidze S., Shekhter A., Khanin A., Pekshev A., Pankratov V. (2004). Clinical evaluation of intraoperative application of air-plasma flow enriched by nitrogen monoxide in operations on the uterus and adnexa. Probl. Gynecol. Obstet. Perinatol..

[17] Grigor’ian A., Grudianov A., Frolova O., Antipova Z., Erokhin A., Shekhter A., Pekshev A. (2001). Use of a new biological factor–exogenous nitric oxide–during surgical treatment of periodontitis. Stomatologiia.

[18] Keidar M. (2015). Plasma for cancer treatment. Plasma Sources Sci. Technol..

[19] Bogaerts A., Khosravian N., Van der Paal J., Verlackt C.C., Yusupov M., Kamaraj B., Neyts E.C. (2015). Multi-level molecular modelling for plasma medicine. J. Phys. D Appl. Phys..

[20] Lin A., Gorbanev Y., De Backer J., Van Loenhout J., Van Boxem W., Lemière F., Cos P., Dewilde S., Smits E., Bogaerts A. (2019). Non-thermal plasma as a unique delivery system of short-lived reactive oxygen and nitrogen species for immunogenic cell death in melanoma cells. Adv. Sci..

[21] Lee M.H., Park B.J., Jin S.C., Kim D., Han I., Kim J., Hyun S.O., Chung K.H., Park J.C. (2009). Removal and sterilization of biofilms and planktonic bacteria by microwave-induced argon plasma at atmospheric pressure. New J. Phys..

[22] Leduc M., Guay D., Leask R., Coulombe S. (2009). Cell permeabilization using a non-thermal plasma. New J. Phys..

[23] Sato T., Ochiai S., Urayama T. (2009). Generation and transport mechanisms of chemical species by a post-discharge flow for inactivation of bacteria. New J. Phys..

[24] Nosenko T., Shimizu T., Morfill G. (2009). Designing plasmas for chronic wound disinfection. New J. Phys..

[25] Rossi F., Kylián O., Rauscher H., Hasiwa M., Gilliland D. (2009). Low pressure plasma discharges for the sterilization and decontamination of surfaces. New J. Phys..

[26] Baxter H.C., Richardson P.R., Campbell G.A., Kovalev V.I., Maier R., Barton J.S., Jones A.C., DeLarge G., Casey M., Baxter R.L. (2009). Application of epifluorescence scanning for monitoring the efficacy of protein removal by RF gas–plasma decontamination. New J. Phys..

[27] Grundmann H., Aires-de Sousa M., Boyce J., Tiemersma E. (2006). Emergence and resurgence of meticillin-resistant *Staphylococcus aureus* as a public-health threat. Lancet.

[28] Morfill G., Shimizu T., Steffes B., Schmidt H. (2009). Nosocomial infections—A new approach towards preventive medicine using plasmas. New J. Phys..

[29] Klein L.L., Gibbs R.S. (2004). Use of microbial cultures and antibiotics in the prevention of infection-associated preterm birth. Am. J. Obstet. Gynecol..

[30] Fluhr J.W., Sassning S., Lademann O., Darvin M.E., Schanzer S., Kramer A., Richter H., Sterry W., Lademann J. (2012). In vivo skin treatment with tissue-tolerable plasma influences skin physiology and antioxidant profile in human stratum corneum. Exp. Dermatol..

[31] Etufugh C.N., Phillips T.J. (2007). Venous ulcers. Clin. Dermatol..

[32] Potter M.J., Harrison R., Ramsden A., Bryan B., Andrews P., Gault D. (2007). Facial acne and fine lines: Transforming patient outcomes with plasma skin regeneration. Ann. Plast. Surg..

[33] Hanash S.M., Pitteri S.J., Faca V.M. (2008). Mining the plasma proteome for cancer biomarkers. Nature.

[34] Yuan Y., Liu D., Xiang R., Li Z., Zhang M., Zhao J., Fan B., Li C., Niu D., Ren J. (2021). Advances in biodegradation of macrolide antibiotics. Sheng Wu Gong Cheng Xue Bao Chin. J. Biotechnol..

[35] Chen J., Ren Q., Ding Y., Xiong C., Guo W. (2021). Synthesis of bifunctional composites Ag/BiOCl/diatomite: Degradation of tetracycline and evaluation of antimicrobial activity. J. Environ. Chem. Eng..

[36] Xu H., Wang L., Sun J., Wang L., Guo H., Ye Y., Sun X. (2022). Microbial detoxification of mycotoxins in food and feed. Crit. Rev. Food Sci. Nutr..

[37] Suzuki T., Hidaka T., Kumagai Y., Yamamoto M. (2020). Environmental pollutants and the immune response. Nat. Immunol..

[38] Suh H.H., Bahadori T., Vallarino J., Spengler J.D. (2000). Criteria air pollutants and toxic air pollutants. Environ. Health Perspect..

[39] Deblonde T., Cossu-Leguille C., Hartemann P. (2011). Emerging pollutants in wastewater: A review of the literature. Int. J. Hyg. Environ. Health.

[40] Danner M.C., Robertson A., Behrends V., Reiss J. (2019). Antibiotic pollution in surface fresh waters: Occurrence and effects. Sci. Total Environ..

[41] Daughton C.G. (2011). Illicit drugs: Contaminants in the environment and utility in forensic epidemiology. Rev. Environ. Contam. Toxicol..

[42] da Rocha M.E.B., Freire F.d.C.O., Maia F.E.F., Guedes M.I.F., Rondina D. (2014). Mycotoxins and their effects on human and animal health. Food Control.

[43] Sanito R.C., You S.J., Wang Y.F. (2022). Degradation of contaminants in plasma technology: An overview. J. Hazard. Mater..

[44] Li H., Li T., He S., Zhou J., Wang T., Zhu L. (2020). Efficient degradation of antibiotics by non-thermal discharge plasma: Highlight the impacts of molecular structures and degradation pathways. Chem. Eng. J..

[45] Magureanu M., Mandache N.B., Parvulescu V.I. (2015). Degradation of pharmaceutical compounds in water by non-thermal plasma treatment. Water Res..

[46] Gao J., Wang X., Hu Z., Deng H., Hou J., Lu X., Kang J. (2003). Plasma degradation of dyes in water with contact glow discharge electrolysis. Water Res..

[47] Kim H.H., Teramoto Y., Ogata A., Takagi H., Nanba T. (2016). Plasma catalysis for environmental treatment and energy applications. Plasma Chem. Plasma Process..

[48] Lerouge S., Wertheimer M.R., Yahia L. (2001). Plasma sterilization: A review of parameters, mechanisms, and limitations. Plasmas Polym..

[49] Koomen J.M., Li D., Xiao L.c., Liu T.C., Coombes K.R., Abbruzzese J., Kobayashi R. (2005). Direct tandem mass spectrometry reveals limitations in protein profiling experiments for plasma biomarker discovery. J. Proteome Res..

[50] Ahmed A., Aziz M., Ejaz S.A., Channar P.A., Saeed A., Zargar S., Wani T.A., Hamad A., Abbas Q., Raza H. (2022). Design, synthesis, kinetic analysis and pharmacophore-directed discovery of 3-ethylaniline hybrid imino-thiazolidinone as potential inhibitor of carbonic anhydrase II: An emerging biological target for treatment of cancer. Biomolecules.

[51] Verkhivker G., Agajanian S., Kassab R., Krishnan K. (2022). Integrating Conformational Dynamics and Perturbation-Based Network Modeling for Mutational Profiling of Binding and Allostery in the SARS-CoV-2 Spike Variant Complexes with Antibodies: Balancing Local and Global Determinants of Mutational Escape Mechanisms. Biomolecules.

[52] Sivakumar D., Stein M. (2021). Binding of SARS-CoV covalent non-covalent inhibitors to the SARS-CoV-2 papain-like protease and ovarian tumor domain deubiquitinases. Biomolecules.

[53] Avci F.G., Sariyar Akbulut B., Ozkirimli E. (2018). Membrane active peptides and their biophysical characterization. Biomolecules.

[54] El-Demerdash A., Metwaly A.M., Hassan A., Abd El-Aziz T.M., Elkaeed E.B., Eissa I.H., Arafa R.K., Stockand J.D. (2021). Comprehensive virtual screening of the antiviral potentialities of marine polycyclic guanidine alkaloids against SARS-CoV-2 (COVID-19). Biomolecules.

[55] De Boer D., Nguyen N., Mao J., Moore J., Sorin E.J. (2021). A comprehensive review of cholinesterase modeling and simulation. Biomolecules.

[56] Ghasemitarei M., Ghorbi T., Yusupov M., Zhang Y., Zhao T., Shali P., Bogaerts A. (2023). Effects of nitro-oxidative stress on biomolecules: Part 1—non-reactive molecular dynamics simulations. Biomolecules.

[57] Baig M.W., Pederzoli M., Kỳvala M., Pittner J. (2025). Quantum Chemical and Trajectory Surface Hopping Molecular Dynamics Study of Iodine-Based BODIPY Photosensitizer. J. Comput. Chem..

[58] Wen J., Mai S., González L. (2023). Excited-state dynamics simulations of a light-driven molecular motor in solution. J. Phys. Chem. A.

[59] van Gunsteren W.F., Daura X., Hansen N., Mark A.E., Oostenbrink C., Riniker S., Smith L.J. (2018). Validation of molecular simulation: An overview of issues. Angew. Chem. Int. Ed..

[60] Harrison J.A., Schall J.D., Knippenberg M.T., Gao G., Mikulski P.T. (2008). Elucidating atomic-scale friction using molecular dynamics and specialized analysis techniques. J. Phys. Condens. Matter.

[61] Yusupov M., Dewaele D., Attri P., Khalilov U., Sobott F., Bogaerts A. (2023). Molecular understanding of the possible mechanisms of oligosaccharide oxidation by cold plasma. Plasma Process. Polym..

[62] Li S., Yao X., Wang X., Tian S., Zhang Y. (2022). Reactive molecular dynamics simulation on degradation of aflatoxin B1 by cold atmospheric plasmas. Innov. Food Sci. Emerg. Technol..

[63] Tian S.Q., Wang X.L., Zhang Y.T. (2021). Numerical study on interactions of atmospheric plasmas and vegetable oils by reactive molecular dynamic simulations. Plasma Process. Polym..

[64] Dong Y., Li Q., Martini A. (2013). Molecular dynamics simulation of atomic friction: A review and guide. J. Vac. Sci. Technol. A.

[65] Van Duin A.C., Dasgupta S., Lorant F., Goddard W.A. (2001). ReaxFF: A reactive force field for hydrocarbons. J. Phys. Chem. A.

[66] Russo M.F., Van Duin A.C. (2011). Atomistic-scale simulations of chemical reactions: Bridging from quantum chemistry to engineering. Nucl. Instrum. Methods Phys. Res. Sect. B Beam Interact. Mater. Atoms.

[67] Chenoweth K., Van Duin A.C., Goddard W.A. (2008). ReaxFF reactive force field for molecular dynamics simulations of hydrocarbon oxidation. J. Phys. Chem. A.

[68] Senftle T.P., Hong S., Islam M.M., Kylasa S.B., Zheng Y., Shin Y.K., Junkermeier C., Engel-Herbert R., Janik M.J., Aktulga H.M. (2016). The ReaxFF reactive force-field: Development, applications and future directions. Npj Comput. Mater..

[69] Sun H., Ren P., Fried J. (1998). The COMPASS force field: Parameterization and validation for phosphazenes. Comput. Theor. Polym. Sci..

[70] Nussinov Z., Van Den Brink J. (2015). Compass models: Theory and physical motivations. Rev. Mod. Phys..

[71] Guo J.S., Tian S.Q., Zhang Y.T. (2023). Reactive molecular dynamics simulations on interaction mechanisms of cold atmospheric plasmas and peptides. Phys. Plasmas.

[72] Chai Z.N., Wang X.C., Yusupov M., Zhang Y.T. (2024). Unveiling the interaction mechanisms of cold atmospheric plasma and amino acids by machine learning. Plasma Processes Polym..

[73] Srinivas U.S., Tan B.W., Vellayappan B.A., Jeyasekharan A.D. (2019). ROS and the DNA damage response in cancer. Redox Biol..

[74] Juan C.A., Pérez de la Lastra J.M., Plou F.J., Pérez-Lebeña E. (2021). The chemistry of reactive oxygen species (ROS) revisited: Outlining their role in biological macromolecules (DNA, lipids and proteins) and induced pathologies. Int. J. Mol. Sci..

[75] Cui J., Zhao T., Zou L., Wang X., Zhang Y. (2018). Molecular dynamics simulation of *S. cerevisiae* glucan destruction by plasma ROS based on ReaxFF. J. Phys. D Appl. Phys..

[76] Yao X., Guo J.S., Zhang Y.T. (2022). Unveiling pathways of oxytetracycline degradation induced by cold atmospheric plasma. AIP Adv..

[77] Guo J., Zhang Y. (2023). Reactive molecular dynamics simulation on degradation of tetracycline antibiotics treated by cold atmospheric plasmas. Molecules.

[78] He D., Sun Y., Xin L., Feng J. (2014). Aqueous tetracycline degradation by non-thermal plasma combined with nano-TiO2. Chem. Eng. J..

[79] Wang C., Qu G., Wang T., Deng F., Liang D. (2018). Removal of tetracycline antibiotics from wastewater by pulsed corona discharge plasma coupled with natural soil particles. Chem. Eng. J..

[80] Yuan F., Hu C., Hu X., Wei D., Chen Y., Qu J. (2011). Photodegradation and toxicity changes of antibiotics in UV and UV/H2O2 process. J. Hazard. Mater..

[81] Guo H., Wang Y., Yao X., Zhang Y., Li Z., Pan S., Han J., Xu L., Qiao W., Li J. (2021). A comprehensive insight into plasma-catalytic removal of antibiotic oxytetracycline based on graphene-TiO_2_-Fe_3_O_4_ nanocomposites. Chem. Eng. J..

[82] Yi R., Guo H., Wang H., Du D., Zhang Q., Yi C. (2021). Multiple production of highly active particles for oxytetracycline degradation in a large volume strong ionization dielectric barrier discharge system: Performance and degradation pathways. Sep. Purif. Technol..

[83] Qiao X.X., Liu X.J., Zhang W.Y., Cai Y.L., Zhong Z., Li Y.F., Lü J. (2021). Superior photo–Fenton activity towards chlortetracycline degradation over novel g–C3N4 nanosheets/schwertmannite nanocomposites with accelerated Fe (III)/Fe (II) cycling. Sep. Purif. Technol..

[84] Li S., Wang X., Li L., Liu J., Ding Y., Zhao T., Zhang Y. (2022). Atomic-scale simulations of the deoxynivalenol degradation induced by reactive oxygen plasma species. Food Res. Int..

[85] Appell M., Bosma W.B. (2015). Assessment of the electronic structure and properties of trichothecene toxins using density functional theory. J. Hazard. Mater..

[86] Heirman P., Verswyvel H., Bauwens M., Yusupov M., De Waele J., Lin A., Smits E., Bogaerts A. (2024). Effect of plasma-induced oxidation on NK cell immune checkpoint ligands: A computational-experimental approach. Redox Biol..

[87] Takai E., Kitamura T., Kuwabara J., Ikawa S., Yoshizawa S., Shiraki K., Kawasaki H., Arakawa R., Kitano K. (2014). Chemical modification of amino acids by atmospheric-pressure cold plasma in aqueous solution. J. Phys. D Appl. Phys..

[88] Zhou R., Zhou R., Zhuang J., Zong Z., Zhang X., Liu D., Bazaka K., Ostrikov K. (2016). Interaction of atmospheric-pressure air microplasmas with amino acids as fundamental processes in aqueous solution. PLoS ONE.

[89] Yusupov M., Wende K., Kupsch S., Neyts E.C., Reuter S., Bogaerts A. (2017). Effect of head group and lipid tail oxidation in the cell membrane revealed through integrated simulations and experiments. Sci. Rep..

[90] Yusupov M., Neyts E.C., Verlackt C.C., Khalilov U., Van Duin A.C., Bogaerts A. (2015). Inactivation of the endotoxic biomolecule lipid a by oxygen plasma species: A reactive molecular dynamics study. Plasma Process. Polym..

[91] Tampieri F., Espona-Noguera A., Labay C., Ginebra M.P., Yusupov M., Bogaerts A., Canal C. (2023). Does non-thermal plasma modify biopolymers in solution? A chemical and mechanistic study for alginate. Biomater. Sci..

[92] Feizollahi E., Arshad M., Yadav B., Ullah A., Roopesh M. (2021). Degradation of deoxynivalenol by atmospheric-pressure cold plasma and sequential treatments with heat and UV light. Food Eng. Rev..

[93] He W.J., Zhang L., Yi S.Y., Tang X.L., Yuan Q.S., Guo M.W., Wu A.B., Qu B., Li H.P., Liao Y.C. (2017). An aldo-keto reductase is responsible for Fusarium toxin-degrading activity in a soil Sphingomonas strain. Sci. Rep..

[94] Li Y., Fan L., Tang X.M., Yang D.M., Hu J.H., Wu Y.Z., Zhan S., Yang D.C. (2021). Synthesis and antibacterial activity of C-7 haloacyl cephalosporins. Yao Xue Xue Bao.

[95] Abdul-Mutakabbir J.C., Alosaimy S., Morrisette T., Kebriaei R., Rybak M.J. (2020). Cefiderocol: A novel Siderophore cephalosporin against multidrug-resistant gram-negative pathogens. Pharmacother. J. Hum. Pharmacol. Drug Ther..

[96] Nicolás-Vázquez I., Méndez-Albores A., Moreno-Martínez E., Miranda R., Castro M. (2010). Role of lactone ring in structural, electronic, and reactivity properties of aflatoxin B1: A theoretical study. Arch. Environ. Contam. Toxicol..

[97] Shi H., Cooper B., Stroshine R.L., Ileleji K.E., Keener K.M. (2017). Structures of degradation products and degradation pathways of aflatoxin B1 by high-voltage atmospheric cold plasma (HVACP) treatment. J. Agric. Food Chem..

[98] Wielogorska E., Ahmed Y., Meneely J., Graham W.G., Elliott C.T., Gilmore B.F. (2019). A holistic study to understand the detoxification of mycotoxins in maize and impact on its molecular integrity using cold atmospheric plasma treatment. Food Chem..

[99] Nagy C.M., Fejer S.N., Berek L., Molnar J., Viskolcz B. (2005). Hydrogen bondings in deoxynivalenol (DON) conformations—A density functional study. J. Mol. Struct. THEOCHEM.

[100] Hojnik N., Modic M., Walsh J.L., Žigon D., Javornik U., Plavec J., Žegura B., Filipič M., Cvelbar U. (2021). Unravelling the pathways of air plasma induced aflatoxin B1 degradation and detoxification. J. Hazard. Mater..

[101] Verardo V., Ferioli F., Riciputi Y., Iafelice G., Marconi E., Caboni M.F. (2009). Evaluation of lipid oxidation in spaghetti pasta enriched with long chain n- 3 polyunsaturated fatty acids under different storage conditions. Food Chem..

[102] Zhang W., Watanabe K., Cai X., Jung M.E., Tang Y., Zhan J. (2008). Identifying the minimal enzymes required for anhydrotetracycline biosynthesis. J. Am. Chem. Soc..

[103] Poole L.B. (2015). The basics of thiols and cysteines in redox biology and chemistry. Free Radic. Biol. Med..

[104] Vidossich P., Magistrato A. (2014). QM/MM molecular dynamics studies of metal binding proteins. Biomolecules.

